# Evaluation of flicker induced hyperemia in the retina and optic nerve head measured by Laser Speckle Flowgraphy

**DOI:** 10.1371/journal.pone.0207525

**Published:** 2018-11-28

**Authors:** Klemens Fondi, Ahmed M. Bata, Nikolaus Luft, Katarzyna J. Witkowska, René M. Werkmeister, Doreen Schmidl, Matthias Bolz, Leopold Schmetterer, Gerhard Garhöfer

**Affiliations:** 1 Department of Clinical Pharmacology, Medical University of Vienna, Vienna, Austria; 2 Kepler University Clinic, Johannes Kepler University, Linz, Austria; 3 University Eye Hospital, Ludwig-Maximilians-University, Munich, Germany; 4 Center for Medical Physics and Biomedical Engineering, Medical University of Vienna, Vienna, Austria; 5 Singapore Eye Research Institute, Singapore, Singapore; 6 Lee Kong Chian School of Medicine, Nanyang Technological University, Singapore, Singapore; 7 Ophthalmology and Visual Sciences Academic Clinical Program, Duke-NUS Medical School, Singapore, Singapore; Boston Medical Center, Boston University School of Medicine, UNITED STATES

## Abstract

**Purpose:**

The coupling between neural activity and blood flow is a physiological key principle of ocular blood flow regulation. The current study was performed to investigate whether Laser speckle flowgraphy (LSFG), a commercially available technique for measuring blood flow, is capable to assess flicker-induced haemodynamic changes in the retinal and optic nerve head (ONH) circulation.

**Methods:**

Twenty healthy subjects were included in this cross sectional study. A commercial LSFG instrument was used to measure blood flow at the ONH as well as in retinal vessels before and during stimulation with flickering light. Mean blur rate (MBR), a measure of relative blood flow velocity, was obtained for the ONH and relative flow volume (RFV) a measure of relative blood flow of the respective retinal vessels.

**Results:**

Stimulation with flicker light increased ONH MBR by +17.5%±6.6% (p<0.01). In retinal arteries, flicker stimulation led an increase of +23.8±10.0% (p<0.05) in total RFV. For retinal veins, an increase of +23.1%±11.0 (p<0.05) in total RFV was observed during stimulation. A higher response was observed in nasal RFV compared to temporal RFV in retinal arteries (nasal: +28.9%±20.0%; temporal: +20.4%±17.6%, p<0.05) and veins (nasal: +28.3%±19.6%; temporal +17.8%±18.9%, p<0.05).

**Conclusion:**

As shown previously with other techniques, flicker stimulation leads to an increase in retinal and optic nerve head blood flow. Our results indicate that LSFG is an appropriate method for the quantification of retinal and ONH blood flow during visual stimulation and may be used as a non-invasive, easy to use tool to assess neuro-vascular coupling in humans.

## Background

Neuro-vascular coupling is a physiological key feature of neural tissues to adapt blood flow to local metabolic demands. Starting with the hypothesis described in the landmark paper of Roy and Sherington more than 100 years ago,[1] it was proven that neural tissue is capable to increase local perfusion in correspondence to its current work-load with high local and temporal resolution.[2–4] This vascular response—also termed functional hyperemia or neuro-vascular coupling[2, 4]—ensures adequate supply of blood glucose and oxygen in dependence on the local activity and energy consumption of the firing neurons.[5] In this context, it has been shown that breakdown of the coupling between neural activity and blood flow is an early event in several ocular diseases[6–10] and may be a biomarker to identify risk patients or to monitor disease progression.[11, 12]

This approach is currently limited by the fact that the assessment of ocular blood flow is still challenging and no gold standard for the measurement of ocular hemodynamics is currently available.[13] As such, the majority of studies investigating neuro-vascular coupling in humans have used retinal vessel diameter to assess flicker-induced hyperemia.[14–16] Although retinal vessel diameters are a major determent of retinal blood flow, the lack of velocity information does not allow for the quantification of volumetric blood flow, which limits the interpretation of the results.

Recently new techniques have been developed to overcome these limitations. In particular, functional optical coherence tomography (OCT) has been used to assess neuro-vascular coupling in-vivo. As such, studies using multifunction OCT angiography show that this technique allows for the assessment of flicker evoked hemodynamic responses of individual layers of the retina.[17, 18] Alternatively, dual-beam bidirectional Doppler Fourier-domain has been used to quantify flicker evoked hemodynamic changes in both animal and human experiments.[19, 20] However, the latter techniques are currently not commercially available and therefore only limited to specialized research centers.

Beside these new approaches, Laser speckle flowgraphy (LSFG) has gained much interest for the assessment of ocular perfusion in humans.[21, 22] [23–25] The fact that LSFG is a non-invasive and FDA approved method for the assessment of retinal blood flow with short measuring times makes it an attractive approach for the assessment of neuro-vascular coupling in humans. In addition, the LSFG technique offers the possibility to assess changes in blood flow with high temporal and spatial resolution.

In the current study we tested the hypothesis that LSFG is a tool to investigate neuro-vascular coupling in humans. Further, we set out to investigate whether there are spatial differences in the hyperemic response to visual stimulation.

## Methods

### Subjects

Twenty healthy subjects were included in this study. The study protocol was approved by the Ethics Committee of the Medical University of Vienna and the study was performed in adherence to the guidelines of the Declaration of Helsinki as well as Good Clinical Practice guidelines. Subjects were selected by the Department of Clinical Pharmacology at the Medical University of Vienna and written informed consent was obtained from all study participants.

All subjects passed a screening examination including physical examination, blood pressure measurement and ophthalmic examination comprising best-corrected visual acuity testing using standard Early Treatment of Diabetic Retinopathy Study (ETDRS) charts, slit-lamp examination including indirect funduscopy, measurement of intraocular pressure (IOP) using Goldmann applanation tonometry. Exclusion criteria were age < 18 years, pregnancy or lactation, smoking, ametropia > 3 dpt, presence of any ocular pathologies, systemic hypertension (defined as either systolic blood pressure > 140 mmHg or diastolic blood pressure > 90 mmHg, or a diagnosis of systemic hypertension in the medical history), history or family history of epilepsy, clinically relevant illness prior to the study, as well as participation in a clinical study, blood donation or intake of any medication in the 3 weeks before the study. Pupil dilation was achieved by one drop of 0.5% tropicamide (Mydriaticum “Agepha”, Agepha, Vienna, Austria). Measurements were performed after a resting period of 20 minutes in ambient light conditions to achieve stable hemodynamic conditions and to allow for equal light adaptation for all subjects under study. In all subjects, measurements were performed in the right eye. Measurements of intraocular pressure (IOP) and systemic hemodynamics were done before and after the LSFG measurements.

### Blood pressure measurement

Systolic, diastolic and mean blood pressures (MAP) were measured on the upper arm by an automated oscillometric device (HP-CMS patient monitor, Hewlett Packard, Palo Alto, Calif., USA).

### Laser speckle flowgraphy

A commercially available LSFG system (LSFG-NAVI; Softcare Co., Ltd., Fukuoka, Japan) was used to measure perfusion in a custom-built model eye and in retinal vessels in vivo before and during flicker stimulation. The principles of LSFG have been described in detail previously.[26–28] Briefly, the technique is based on the observation that if an irregular surface is illuminated with a coherent light source the backscattered light gives the appearance of a consistent scatter pattern. Moving corpuscular blood components such as erythrocytes cause a distinct variation in the speckle pattern, becoming manifest in a reduction of speckle contrast and variation, caused by integration of dynamic speckle during camera exposure time. As described in more detail below, flow information is generated based on statistical analysis of the above described speckle pattern, which is recorded by a digital camera.

The LSFG device used in this study comprised a fundus camera supplied with an 830-nm diode laser and a digital charge-coupled device camera (750 x 360 pixels). The principal output parameter of LSFG, mean blur rate (MBR), is a parameter representing a measurement of relative blood flow velocity and is expressed in arbitrary units (au). During a single LSFG scan, a total of 118 images are continuously recorded at a rate of 30 frames per second over a time period of approximately 4 seconds. The device is equipped with an analysis software (LSFG Analyzer version 3.1.58; Softcare), which synchronizes and mathematically averages all 118 acquired images to produce a so-called ‘‘composite map” showing the distribution of perfusion in the ocular fundus within one cardiac cycle.

Quantification of blood flow of the LSFG system used is based on first order statistics using the standard deviation of the intensity distribution of the speckle pattern (*δ*). This equals to the mean intensity <*I*> under idealized conditions, but may be lower in reality du to inhomogeneities in the scattering tissue. For mapping blood flow space–time correlation function of the speckle intensity fluctuation is calculated. The reciprocal of speckle contrast *δ*/<*I*> is dependent on the ratio of the correlation time of the temporal fluctuations in intensity (*τ_c_*) and exposure time (*T*) and

Speckle contrast is then calculated as follows:

$$c\frac{\delta }{<I>}\infty \sqrt{\frac{\tau_{c}}{2T}\{1-exp(\frac{-2T}{\tau_{c}})\}}$$

where c is a constant dependent on exposure time. The normalized blur (NB) which is an approximation of the reciprocal of speckle contrast. In the present system velocity is calculated based on square blur rate (SBR). In case of a CCD array the SBR can be approximated as

$${SBR}_{n,m,t}={<I_{n,m,t}>}^{2}/{(<I_{n,m,t}^{2}>-<I_{n,m,t}>}^{2})$$

where where I_n,m,t_ is the intensity detected at the pixel (n,m), is integrated over the exposure time. The bracket <> denotes average over 32 frames of the integrated light intensity[29]. The MBR calculated as a running average over 26 pixels and proportional to SBR.

### In vitro capillary measurements

In vitro measurements were performed to exclude that flicker light (see below) itself influences the measurements with the LSFG device. For this purpose, a model eye with a flow phantom was utilized as described previously.[25] Briefly, the model eye consisted of an artificial pupil with a diameter of 4.0 mm and a lens with a focal length of 30.0 mm. A glass capillary with an inner diameter of 300 μm was placed in the focal plane of this model eye to serve as a flow phantom. For the simulation of blood flow within the capillary, a constant and continuous flow of diluted milk was supplied by an infusion pump (Pilot C model; Fresenius, Homburg, Germany). LSFG measurements were performed three successive times at 17 different flow velocities in the range of 0.5 mm/s to 47 mm/s under baseline conditions as well as under diffuse luminance flicker illumination. The mean value of the three consecutive measurements was used for comparison with the predefined flow velocities (MFV).

### Assessment of optic nerve head blood flow

**Fig 1:**
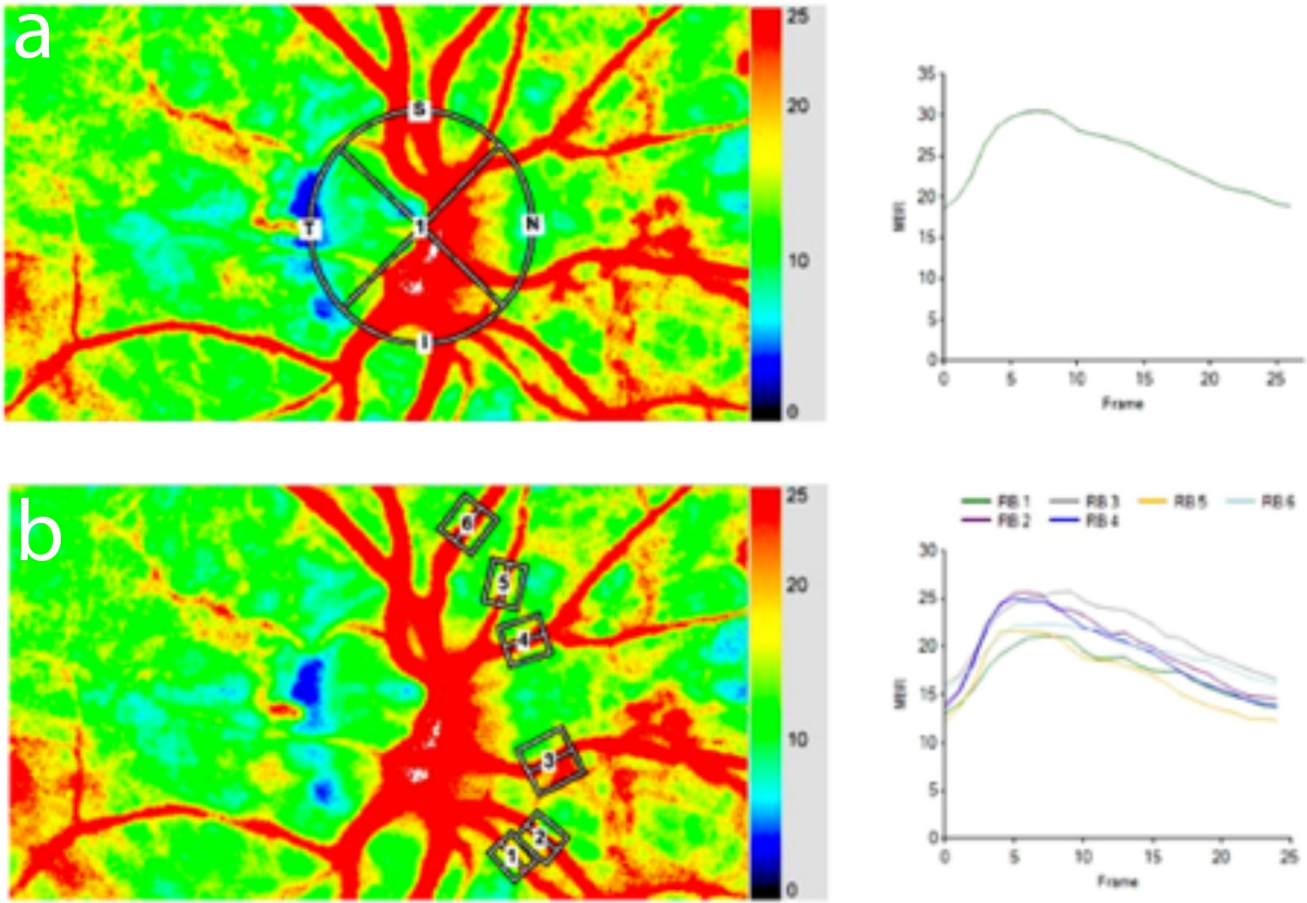
LSFG composite map with regions of interest (ROI) around the optic nerve head (a) and marking all nasal vessels (b). The corresponding flow diagram depicting MBR over recorded frame is shown on the right side of the figure.

For analysis of blood flow the ONH area was manually delineated by positioning an ellipsoid region of interest at the ONH margin on the color-coded map using the built-in software (Fig 1). MBR, which represents the mean blur rate of the whole optic nerve head area, was used to assess blood flow of the whole ONH area. Then, tissue areas within the ONH area were automatically detected by the built-in software. By subtracting the tissue area from MBR, the perfusion of the major vessels within the optic nerve head area (MV) is calculated by the LSFG software.

### Assessment of retinal blood flow

To assess flicker responses in the retinal circulation the so called “relative flow volume” (RFV) has been used. This recently introduced parameter[30] represents the primary output parameter of LSFG for the analysis of retinal perfusion. This index of blood flow in retinal vessels is automatically calculated by subtracting the background MBR in the nonvessel area from the MBR detected within the retinal vessel area and has been shown to accurately indicate relative retinal perfusion.[25, 30] For this purpose, rectangular band is centered on the retinal vessel of interest on the color-coded map of the fundus, using the built-in image analysis software. Within the selected area, the retinal vessel is then automatically allocated by computing a threshold between MBR values in the retinal vessel and the background MBR originating from perfusion of the underlying choroid.[30] For subgroup analysis, retinal arteries and veins were grouped into “nasal” and “temporal” based on their anatomical location with respect to the ONH (Fig 1).

### Diffuse luminance flicker stimulation experimental paradigm

A custom-build external illumination unit, comprising a white light-emitting diode (LED) and a control module, was used to apply diffuse luminance flicker stimulation. The LED was attached to the external fixation target and placed between the LSFG device and the eye next to the lens of the camera, to achieve sufficient flicker illumination of the retina without impeding the LSFG measurements. The frequency of the diffuse luminance flicker stimuli was set to 12 Hz and produced a square wave light pattern generated by the control module of the flicker device with a stimulation depth of 100%. The LED produced a luminance of approximately 10000 cd/m^2^ on the ocular surface, which is well below the maximum permissible exposure (MPE) as specified by the international guidelines for save use of lasers [31], to ensure the safety of the eye.

### *In vivo* experimental paradigm and analysis

After a short resting period of 20 minutes to obtain stable hemodynamic condition, three baseline LSFG measurements were performed. All subjects were measured under light adapted conditions. Diffuse luminance flicker illumination was applied for 60 seconds and three consecutive LSFG measurements lasting approximately 4 seconds were performed at the same position as the baseline measurements during flicker stimulation at the end of the 60 second flicker period. Identical position and size of the bands was maintained in all subsequent scans of the same subject using the “follow up scan” function of the software.

### Statistical analysis

All statistical analyses were done using the Statistica software package (Release 6.0, StatSoft Inc., Tulsa, OK, USA). Baseline characteristics of systemic hemodynamic variables and intraocular pressure were analyzed using descriptive statistics. Flicker-induced changes (FL) in ocular hemodynamic parameters are expressed as percentage change over baseline (BL) values, that is (FL−BL) × 100/BL. Two-tailed paired t-tests were used to determine statistical significance of flicker induced hyperemia and other dependent variables. Two-tailed unpaired t-test was used to compare flicker responses between temporal and nasal vessels. Linear regression analysis was used to assess correlation between glass capillary measurements with and without flicker stimulation. All results are presented as means ± standard deviation. A P<0.05 or smaller was considered as level of significance.

## Results

### In vitro experiments

**Fig 2:**
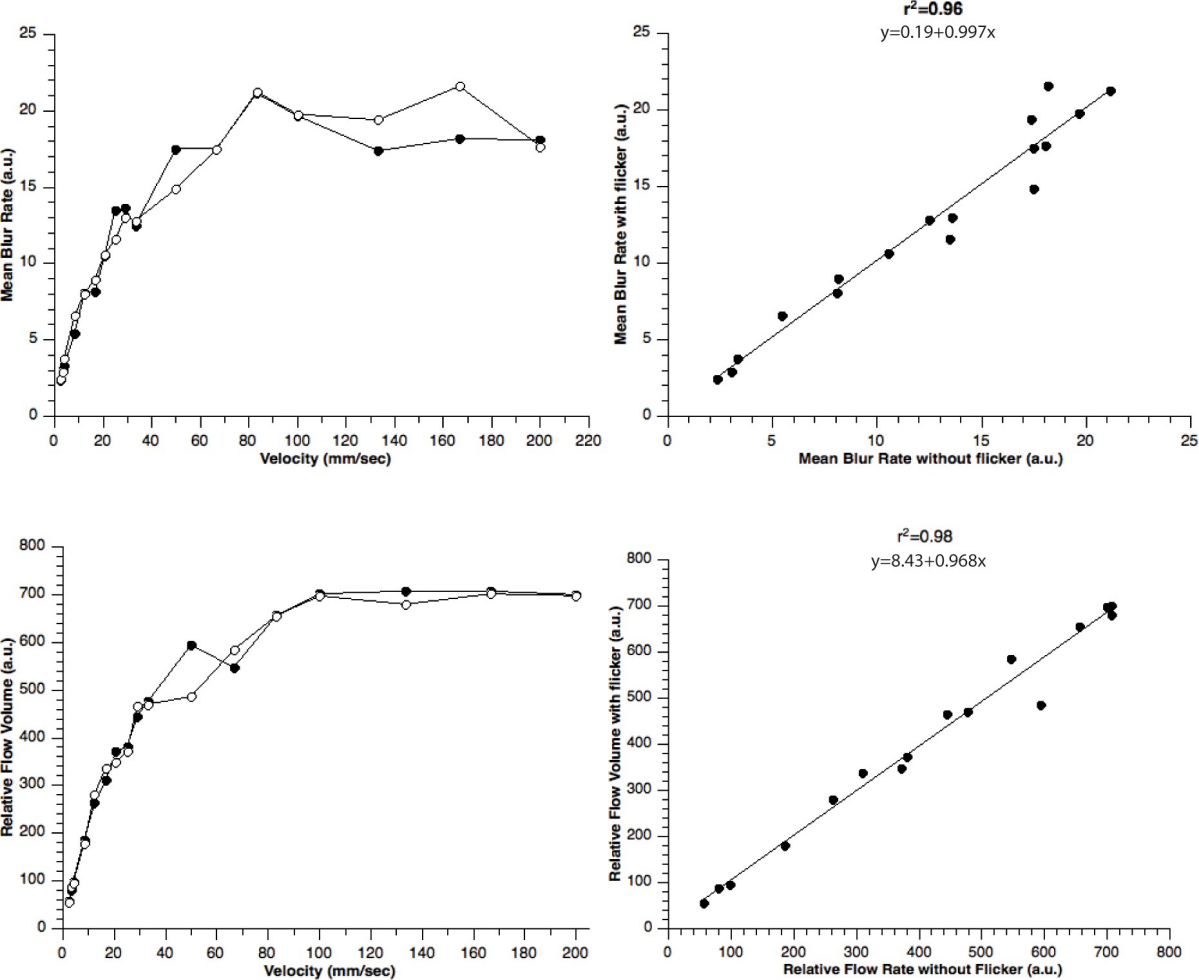
In vitro experiment: The relationship between preset velocity rates in the glascapillary and LSFG measurements in the present (black circles) and absence (open circles) of flicker stimulation (left panels). Right panels show the linear correlation between LSFG measurements in presence and absence of flicker stimulation.

The relationship between pre-set velocity of diluted milk in the glass capillary and LSFG measurements in the presence and absence of flicker stimulation is shown in Fig 2. As shown in the left panels of Fig 2, mean blur rate was found to saturate at approximately 20 au and relative flow volume at approximately 650 au. Below these saturation values, there was a linear relationship between MBR and velocity (r^2^ = 0.91, p<0.05) and MFV and velocity (r^2^ = 0.96, p<0.05), respectively. Further, linear regression analysis shows a good correlation between velocity rates measured in the glass capillary with and without flicker stimulation for MBR (r^2^ = 0.96, p<0.05) and for MFV (r^2^ = 0.98, p<0.05), indicating that flicker stimulation has no influence on the measurements (Fig 2).

### In vivo experiments

A total of 20 healthy subjects (11 female; 9 male) were included in the study. The average age of the participating subjects was 25±7 years, MAP was 91±8 mmHg and intraocular pressure was 15mmHg±1.6mmHg.

At the optic nerve head, stimulation with flicker light increased total MBR significantly by +17.5%±6.6% (p<0.01) and MV by +9.4%±10.9% (p<0.01) respectively. In retinal arteries, flicker stimulation led to an increase of +23.8±10.0% (p<0.05) in total RFV. For retinal veins, an increase of +23.1%±11.0 (p<0.05) in total RFV was observed in response to stimulation with flicker light. Absolute data for optic nerve head MBR and retinal RFV is described in Table 1.

**Table 1:** Optic nerve head mean blur rate (MBR) and relative flow volume (RFV) in retinal arteries and veins at baseline and during flicker stimulation (n = 20). Data is presented as mean± SD.

	Baseline	Flicker Stimulation
Optic Nerve Head MBR (a.u.)	24.6±4.0	29.0±5.7
Retinal arteries RFV (a.u.)	202±39	249±48
Retinal veins RFV (a.u.)	258±44	319±44

**Fig 3:**
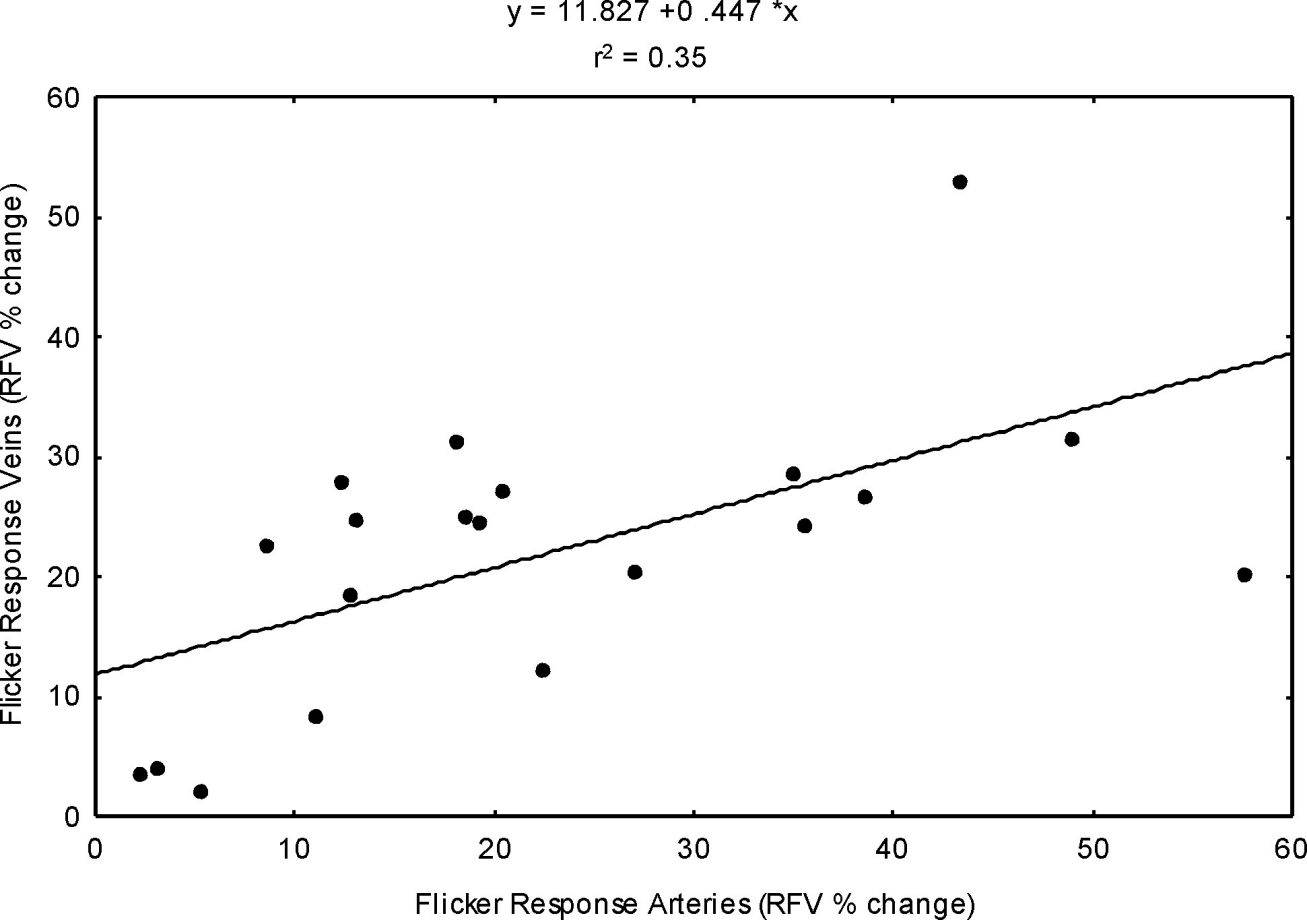
The correlation between flicker induced hyperemia in retinal arteries and retinal veins expressed as percent change over baseline (n = 20, p<0.05).

As shown in Fig 3, there was a significant correlation between flicker induced hyperemia in retinal arteries and the flicker response in retinal veins (Fig 3, r^2^ = 0.35, p<0.05).

Subgroup analysis comparing the flicker response of temporal and nasal retinal arteries revealed a slightly higher response in nasal (+28.9%±20.0%) RFV compared to temporal RFV (+20.4%±17.6%, p<0.05). Consistent with the arterial data, flicker stimulation led to a more pronounced increase in RFV (+28.3%±19.6%) in nasal veins compared to temporal vessels (+17.8%±18.9%, p<0.05).

## Discussion

Our experiments show for the first time that LSFG is a fast and easy to use technique to assess flicker light induced hyperemia of the posterior ocular pole in humans. In keeping with previous results using other techniques for the assessment of ocular blood flow, our data show a pronounced increase in retinal and optic nerve head blood flow during visual stimulation with flickering light. Further, in contrast to other methods for the assessment of retinal hemodynamics, our results indicate that the LSFG technique used in the current study is also capable to gain information on the flicker induced hyperemic response at different locations on the retina with high spatial and temporal resolution.

There is compelling evidence using a variety of different technical approaches that in the retina, stimulation with flickering light leads to an immediate vasodilatation of the retinal vasculature[32] and an increase in retinal blood flow [33] as well as an hyperemic response in the optic nerve head.[34] Using a combination of bi-directional laser Doppler flowmetry and fundus camera based measurement of retinal vessel calibers, studies have shown that stimulation with flickering light increases retinal blood flow in the range of 40% to 60%.[33, 35, 36]

Comparing our data to these previous results as well as Doppler OCT studies[20], flicker induced hyperemic responses found in the current study are considerably lower. The reason for this difference is not entirely clear, but may be due to the several technical differences between the methods used for blood flow measurement: First, although a good correlation of LSFG data with absolute blood flow as obtained from Doppler OCT was found, the data revealed a positive zero offset compared to Doppler OCT measurements.[25] We have hypothesized in this previous report that the zero offset may be due to an underestimation of blood flow in higher flow velocity ranges. This seems reasonable since a clear saturation effect has been found in the referenced work [25], which is also reflected in the data of the current study (Fig 2). These findings indicate that a maximum velocity threshold exists that can be detected with the LSFG technique. Thus, we cannot fully exclude that strong increases in velocity as they might occur during flicker stimulation are not fully reflected in the LSFG data because of mentioned saturation effect. Previous studies have reported blood flow velocities of approximately 5-20mm/sec in major retinal vessels.[33, 37] Considering an estimated maximum increase of 50% due to flicker stimulation, this may in some cases well exceed the detectable range of the LSFG technique since a saturation effect was seen at 23.5mm/sec in vitro.[25] We did, however, observe comparable flicker responses in both retinal arteries and veins indicating for good validity of the measurements.

Along this line of thought, the correlation analysis between retinal arteries and veins of individual patients show a correlation of r^2^ = 35. This is less than one would expect given the good reproducibility of the LSFG system.[25] The reason for this finding is unclear but may be related to both technical and anatomical reasons. First, distribution and number of retinal vessels shows a huge variation between subjects. Thus, retinal arteries and retinal veins do not necessarily supply the same anatomical area of the retina. Consequently, flicker response might not be completely identical and depend on vessel size and number. Further, as stated above, high blood flow velocities in retinal arteries may lead to a saturation effect and therefore to an underestimation of flow velocities particularly in arteries.

In addition to retinal blood flow, our laser speckle measurements reveal an increase in optic nerve head blood flow in response to flicker stimulation in the range of 20%. This is comparable to previous studies using laser Doppler flowmetry to assess flicker induced hemodynamic changes in the optic nerve head.[38–40] As for the retina, the LSFG technique has been validated for the measurement of optic nerve head blood flow against other techniques.[41] Further, the LSFG technique has successfully been used to investigate pressure autoregulation of the optic nerve head in healthy subjects and patients with vascular risk factors.[42, 43] Our results extend these findings by indicating that LSFG is also a suitable technique to assess flicker induced hyperemia in the optic nerve head.

Although, no human data has been published on flicker light induced hyperemia assessed with the LSFG technique, the laser speckle flowmetry has been previously used in animal experiments to assess neuro-vascular coupling in an in-vivo rat model. The data from the latter experiments are in keeping with our observation that LSGF has the potential to assess neuro-vascular coupling in vivo.[44] In particular, using the LSFG technique, the authors show that the hyperemic response of the retina to visual stimulation with flicker light is dependent on the location of the stimulus relative to the vessel indicating that blood flow in the retina is regulated with high spatial resolution.[44] Applying the laser speckle technique in humans, our results show differences in flicker response between different areas of the retina. Comparing flicker induced hyperemia between temporal and nasal vessels, our data indicate that flicker responses in both retinal arteries and veins are significantly higher in nasal compared to temporal vessels. The reason for this difference is not entirely clear, but may be related to the difference in vessel size in the different regions of the retina. We have previously shown that the diameter response to stimulation with diffuse luminance flicker is larger in vessels with smaller diameters.[45] Thus, our results are in keeping with the latter observation of larger responses in the nasal area of the retina, where vessel diameters are on average smaller compared to the temporal part. In addition, our results show that the inter-individual variability of the measurement is rather high. This is however, in keeping with the results of other studies investigation flicker induced hyperemia, which also show high inter-individual differences.[33, 46] In keeping with the latter results, a recent study investigating total retinal blood by dual-beam bidirectional Doppler Fourier-domain optical coherence tomography during flicker stimulation individual increase in total retinal blood flow during flicker stimulation in the range of 30% and 70%.[20] Although this difference may at least be partially be related to differences in the individual angio-architecture, further research is required to further elucidate this observation.

To exclude that the additional flicker light influences the LSFG measurements, we have performed in-vitro experiments measuring fluid velocities in glass capillaries in the presence and absence of flickering light. As shown in Fig 2, our in-vitro data indicate that there is an excellent association between velocity as assessed with LSFG and the preset fluid velocity in the presence and the absence of flicker light stimulation. Further, there is a good correlation between measurements with and without flicker stimulation (Fig 2). This finding conforms findings that LSFG is a valid method to assess retinal blood flow [25] and it is not influenced by the additional flicker stimulation during the measurements.

Beside the latter findings indicating validity and good reproducibility of the technique, LSFG has several limitations that warrant further discussion.[47] With quantification of LSFG using first order statistics based on SBR or MBR it is not clear whether velocity or flow is measured. The loss of contrast in a speckle pattern depends on the relation between static and dynamic scatterers[48]. The velocity distribution will also determine the contrast in the speckle pattern. The distribution in ocular microvessels is unknown and may differ between different vascular beds. As such MT is likely neither directly proportional to velocity nor to flow. LSFG has, however, been validated against invasive ONH blood flow measurements using hydrogen clearance[41] or fluorescence microspheres[49] technique.

Further, as the main outcome measures RFV and MBR are given in arbitrary units, direct comparisons between patients are difficult.[26] Consequently, comparisons in our study have been performed only in the same tissue type (i.e. retina or optic nerve head). In addition, experimental data from a non-human primate animal model indicates a good and linear correlation between measurements between blood flow as measured with microspheres and LSFG in the optic nerve head[49], which is also in keeping with the in-vitro data of our study.

Further, due to the fact that the instrument uses a 830nm light source, we cannot fully exclude that when measuring hemodynamics of retinal vessels, some of the signal raises from the choroid. However, in the current study the so called “relative flow volume” has been used to assess retinal perfusion. This index of blood flow in retinal vessels is automatically calculated by subtracting the background MBR in the non-vessel area from the MBR detected within the retinal vessel area[30]. By subtracting the background noise from the MBR within the retinal vessels, this would also correct for a potential influence of the choroid. This is in keeping with previous data indicating that RFV accurately reflects relative retinal perfusion[25, 30] in humans.

In conclusion, our results endorse LSGF as a promising and easy to use method to assess neuro-vascular coupling in the human retina as well as in the optic nerve head. Our data indicate that the system also allows to gain insight on hyperemic responses with high spatial resolution, which may offer new possibilities to assess neuro-vascular coupling in different areas of the retinal and in patients with retinal pathologies.

## Supporting information

S1 Dataset This file contains the data underlying the findings of this study. (PDF)

S2 Dataset This file contains the data underlying the findings of this study. (PDF)
