# Supplementary material for: Evaluation of flicker induced hyperemia in the retina and optic nerve head measured by Laser Speckle Flowgraphy
Source: PLoS One. 2018 Nov 28;13(11):e0207525. doi: 10.1371/journal.pone.0207525 (PMC6261588; doi:10.1371/journal.pone.0207525)
Supplement: S1 Dataset — (PDF) [file pone.0207525.s001.pdf]

| Subject No. | Age | MAP | IOP | RFV % change arteries |
|---|---|---|---|---|
| 1 | 21 | 85 | 15 | 0,169025 |
| 2 | 23 | 90 | 16 | 0,269163 |
| 3 | 20 | 71 | 14 | 0,200307 |
| 4 | 31 | 104 | 16 | 0,384577 |
| 5 | 20 | 92 | 17 | 0,059723 |
| 6 | 20 | 87 | 15 | 0,200502 |
| 7 | 23 | 99 | 14 | 0,313442 |
| 8 | 25 | 96 | 13 | 0,330922 |
| 9 | 43 | 94 | 11 | 0,256425 |
| 10 | 26 | 90 | 15 | 0,17148 |
| 11 | 21 | 86 | 16 | 0,191783 |
| 12 | 24 | 76 | 13 | 0,208944 |
| 13 | 37 | 98 | 17 | 0,389236 |
| 14 | 23 | 81 | 17 | 0,263755 |
| 15 | 26 | 91 | 15 | 0,283562 |
| 16 | 21 | 99 | 15 | 0,144603 |
| 17 | 21 | 96 | 16 | 0,356351 |
| 18 | 36 | 92 | 16 | 0,185495 |
| 19 | 24 | 91 | 13 | 0,033974 |
| 20 | 20 | 92 | 15 | 0,348798 |

| RFV % change veins | MBR % change ONH | MV % change ONH |
|---|---|---|
| 0,188416 | 0,148541 | 0,084044 |
| 0,173162 | 0,180804 | 0,068493 |
| 0,192185 | 0,065385 | -0,09381 |
| 0,115031 | 0,176471 | 0,132037 |
| 0,10759 | 0,070909 | 0,142716 |
| 0,144998 | 0,161085 | 0,039106 |
| 0,372418 | 0,198819 | 0,163617 |
| 0,299158 | 0,13628 | 0,140167 |
| 0,297277 | 0,25 | -0,02439 |
| 0,179032 | 0,15213 | 0,106707 |
| 0,202682 | 0,100939 | 0,058491 |
| 0,196944 | 0,177928 | 0,082669 |
| 0,402122 | 0,227806 | 0,135542 |
| 0,190245 | 0,230769 | 0,160041 |
| 0,155871 | 0,159251 | 0,082 |
| 0,40553 | 0,142857 | 0,028643 |
| 0,327692 | 0,238189 | 0,111691 |
| 0,122612 | 0,127168 | 0,00582 |
| 0,114748 | 0,196594 | -0,007115 |
| 0,428333 | 0,351551 | 0,454672 |
