# Supplementary material for: Evaluation of flicker induced hyperemia in the retina and optic nerve head measured by Laser Speckle Flowgraphy
Source: PLoS One. 2018 Nov 28;13(11):e0207525. doi: 10.1371/journal.pone.0207525 (PMC6261588; doi:10.1371/journal.pone.0207525)
Supplement: S2 Dataset — (PDF) [file pone.0207525.s002.pdf]

Vessel Number	Location nasal/temporal	Response arteries %	Vessel Number	Location nasal/temporal	Response veins %
1	N	31,6644114	1	N	22,9037704
2	N	22,4309392	2	N	18,2848508
3	N	19,0705128	3	N	29,7900262
4	N	26,8388106	4	N	43,4824435
5	N	37,4833259	5	N	7,19748088
6	N	19,4470925	6	N	16,3517442
7	N	36,5114975	7	N	22,3381295
8	N	-3,96959459	8	N	40,6671398
9	N	9,0784044	9	N	15,0468673
10	N	73,0138714	10	N	11,5696888
11	N	35,5932203	11	N	19,5839675
12	N	12,9697863	12	N	26,0508309
13	N	30,2992519	13	N	3,1671159
14	N	23,8872404	14	N	20,959596
15	N	16,141929	15	N	22,5165563
16	N	8,44200412	16	N	40,166205
17	N	31,6847826	17	N	90,625
18	N	26,8546904	18	N	31,9772942
19	N	24,3760399	19	N	27,6662484
20	N	44,9587825	20	N	33,8809785
21	N	24,8385708	21	N	26,8589574
22	N	39,9182561	22	N	26,5669516
23	N	85,3083434	23	N	20,087146
24	N	31,475029	24	N	41,4060173
25	N	55,8689718	25	N	19,305136
26	N	81,5384615	26	N	50,524541
27	N	-16,7305236	27	N	11,5107914
28	N	13,2817153	28	N	16,0591844
29	N	11,7567568	29	N	10,8626198
30	N	26,6998342	30	N	11,5942029
31	N	14,3437863	31	N	16,8235776

32	N	27,8927203	32	N	24,1982507
33	N	29,9284579	33	N	19,5272932
34	N	6,73194614	34	N	12,8702758
35	N	16,886931	35	N	23,0421687
36	N	21,7263652	36	N	16,4154104
37	N	55,8648111	37	N	14,5816733
38	N	30,0133988	38	N	33,9665164
39	N	38,0872483	39	N	27,3058252
40	N	37,1239912	40	N	49,7879109
41	N	80,1536492	41	N	52,991453
42	N	16,1794977	42	N	51,8461538
43	N	37,9543533	43	N	16,944539
44	N	48,9701339	44	N	38,9428919
45	N	26,744868	45	N	10,1295097
46	N	17,1805131	46	N	8,2979326
47	N	33,2962757	47	N	20,5251479
48	N	-13,1578947	48	N	42,0220412
49	N	35,1435705	49	N	71,0610932
50	N	31,8011257	50	N	42,8299492
51	N	38,0952381	51	N	29,3776371
52	N	35,5455003	52	N	42,6564068
53	N	27,2302662	53	N	41,2246351
54	N	42,9880843	54	N	-5,19480519
55	N	48,0125523	55	N	26,8368942
56	N	22,7308603	56	N	14,6827043
57	N	26,4813844	57	N	25,1839276
58	N	39,394958	58	N	-0,961538462
59	N	11,5698512	59	N	23,106383
60	N	-0,76754386	60	N	10,4918033
61	N	-3,66259711	61	N	48,6227824
62	N	17,8116002	62	N	107,566462
63	N	43,4138077	63	N	45,1770452

64	T	2,94117647	64	T	13,0863358
65	T	20,4663212	65	T	20,5592105
66	T	35,7969724	66	T	15,0334514
67	T	12,419114	67	T	23,1821454
68	T	0,468018721	68	T	-39,0430622
69	T	9,03328051	69	T	35,0352766
70	T	10,325176	70	T	38,8239014
71	T	51,7034068	71	T	11,3812726
72	T	16,0762943	72	T	38,5982231
73	T	15,638412	73	T	6,70170828
74	T	31,1518325	74	T	-29,5735901
75	T	32,357473	75	T	-2,48803828
76	T	56,2806673	76	T	0
77	T	7,4759701	77	T	10,3530157
78	T	53,5714286	78	T	-7,61392584
79	T	0	79	T	23,7723214
80	T	-11,9402985	80	T	25,3367394
81	T	-0,69735007	81	T	26,336478
82	T	7,42804085	82	T	-22,6502311
83	T	10,2512563	83	T	18,359375
84	T	19,706499	84	T	30,368932
85	T	36,038961	85	T	24,8905355
86	T	28,0603805	86	T	39,0459364
87	T	29,1099476	87	T	36,1403509
88	T	25,0588235	88	T	41,8362732
89	T	29,2706334	89	T	28,7076271
90	T	21,8379282	90	T	4,33668801
91	T	33,3730277	91	T	34,0336134
92	T	-32,3149236	92	T	21,8492524
93	T	5,53907023	93	T	29,9148211
94	T	34,6194503	94	T	21,7688442
95	T	28,3553875	95	T	17,5975516

96	T	32,8939828
97	T	10,2814259
98	T	16,6851564
99	T	-11,97411
100	T	20,3600514
101	T	38,2865807
102	T	13,3886256
103	T	21,968726
104	T	23,8736406
105	T	30,0076161
106	T	26,2189215
107	T	27,7690123
108	T	2,75761974
109	T	41,8674699
110	T	4,60431655
111	T	22,2222222
112	T	40,3757147
113	T	20,6944444
114	T	7,7099587
115	T	41,0769231
116	T	8,71474804
117	T	35,0292398
118	T	34,8394768
119	T	8,58208955
120	T	25,6410256
121	T	28,3769063
122	T	-15,1646447
123	T	2,46406571
124	T	54,376931
125	T	31,2889813
126	T	48,4523057
127	T	27,8975741

96	T	5,9618442
97	T	17,3633441
98	T	26,5986828
99	T	42,6739927
100	T	10,7652703
101	T	46,1145567
102	T	21,484375
103	T	20,6712433
104	T	5,45517123
105	T	19,5434521
106	T	-1,67095116
107	T	39,3810032
108	T	54,7868623
109	T	-27,7976495
110	T	7,44888023
111	T	39,40285
112	T	27,7227723
113	T	17,9719703
114	T	44,1812136
115	T	26,25
116	T	8,64440079
117	T	0,658978583
118	T	1,76230389
119	T	25,734072
120	T	34,5926497
121	T	2,93398533
122	T	-1,24059386
123	T	13,5416667
124	T	38,8299025
125	T	8,49443468
126	T	8,30889541

128	T	2,14190094
129	T	6,66666667
130	T	3,29438805
131	T	29,1364381
132	T	2,15477997
133	T	7,69230769
134	T	39,1420262
135	T	39,1517129
